# Possible Missing Sources of Atmospheric Glyoxal Part I: Phospholipid Oxidation from Marine Algae

**DOI:** 10.3390/metabo14110639

**Published:** 2024-11-19

**Authors:** Renee T. Williams, Annika Caspers-Brown, Camille M. Sultana, Christopher Lee, Jessica L. Axson, Francesca Malfatti, Yanyan Zhou, Kathryn A. Moore, Natalie Stevens, Mitchell V. Santander, Farooq Azam, Kimberly A. Prather, Robert S. Pomeroy

**Affiliations:** 1 Department of Chemistry and Biochemistry, University of California, La Jolla, San Diego, CA 92093, USA; rwill008@gmail.com (R.T.W.); acaspersbrown@gmail.com (A.C.-B.);; 2 School of Public Health, University of Michigan, Ann Arbor, MI 48109, USA; fazam@ucsd.edu; 3 National Institute of Oceanography and Experimental Geophysics, 34100 Trieste, Italy; 4 Scripps Institution of Oceanography, University of California, La Jolla, San Diego, CA 92093, USA; zhouyyahgy@scsio.ac.cn; 5 State Key Laboratory of Marine Environmental Science, Key Laboratory of the MOE for Coastal and Wetland Ecosystems, School of Life Sciences, Xiamen University, Xiamen 361102, China

**Keywords:** gas chromatography mass spectrometry, liquid chromatography tandem mass spectrometry, solid phase extraction, sea spray aerosols, fatty acids, volatile organic compounds

## Abstract

Background: Glyoxal has been implicated as a significant contributor to the formation of secondary organic aerosols, which play a key role in our ability to estimate the impact of aerosols on climate. Elevated concentrations of glyoxal over remote ocean waters suggests that there is an additional source, distinct from urban and forest environments, which has yet to be identified. Herein, we demonstrate that the ocean can serve as an appreciable source of glyoxal in the atmosphere due to microbiological activity. Methods and Results: Based on mass spectrometric analyses of nascent sea spray aerosols and the sea surface microlayer (SSML) of naturally occurring algal blooms, we provide evidence that during the algae death phase phospholipids become enriched in the SSML and undergo autoxidation thereby generating glyoxal as a degradation product. Conclusions: We propose that the death phase of an algal bloom could serve as an important and currently missing source of glyoxal in the atmosphere.

## 1. Introduction

Glyoxal, the smallest of α-dicarbonyl compounds, is a ubiquitous gas-phase oxidation product of a variety of volatile organic compounds (VOCs) found in the atmosphere. The vast majority of research has focused on elevated glyoxal concentrations in urban environments as a result of vehicle emissions, in addition to other anthropogenic sources including biomass and fossil fuel burning [1,2,3,4,5,6,7,8]. The largest known precursor for glyoxal is isoprene, which is emitted from terrestrial biogenic sources and, to a much lesser extent, from the oxidation of monoterpenes [9,10,11,12,13].

Interest in the origins and levels of glyoxal within the atmosphere lies in its potential contribution to aerosol particles that serve as cloud condensation nuclei, which is one of the most poorly understood topics in atmospheric aerosol research [14]. Given the highly water-soluble nature of glyoxal, it readily uptakes water to form aqueous particles (i.e., aerosols, fog, or cloud droplets) where it can be oxidized [15], form oligomers [16], and react with salts [17] and organics [18,19,20] to form secondary organic aerosols (SOA) [9,21,22,23]. It has been shown that sulfuric acid-catalyzed heterogeneous reactions involving glyoxal and other aldehydes can lead to the growth of newly nucleated nanoparticles [14,24,25,26]. Moreover, aqueous-phase processing of glyoxal can result in a range of light-absorbing molecules [17,18,19,27,28,29,30]. Thus, SOA is one of the greatest points of uncertainty in estimating aerosols’ impact on climate [31,32]. Glyoxal’s relevance to atmospheric chemistry and climate change comes from its role in secondary organic aerosol formation, its involvement in oxidative processes, its impact on the global carbon cycle, and its effects on cloud formation and radiative balance.

In urban areas [6,8,23] and forest environments [10,11] reported glyoxal levels range from a few hundred parts per trillion (pptv; pmol/mol) to low thousands of pptv using ground-based instrumentation [33]. Satellite observations have given similar trends of elevated concentrations over these regions [34,35,36]. A number of marine glyoxal measurements have also been reported, but these values are far less consistent. Coburn et al. (2014) calculated eddy covariance measurements of glyoxal and found average mixing ratios of 43 ± 9 pptv and 32 ± 6 pptv in the Southern and Northern hemispheres, respectively, which were collected during a cruise using fast light-emitting diode cavity-enhanced differential optical absorption spectroscopy (Fast LED-CE-DOAS) [37]. Based on 10 field campaigns across the world’s oceans employing multiaxis (MAX) DOAS and long-path DOAS, similar concentrations of approximately 25 pptv with an upper limit of 40 pptv within the marine boundary layer (MBL) have also been calculated [33]. Most recently, Lawson et al. (2015) noted seasonal in situ mixing ratios of 7 ppt (winter) and 23 ppt (summer) over temperate ocean waters based on 2,4-dinitrophenylhydrazine derivatization of glyoxal and ultraviolet (UV) detection [38]. Although the summer mixing ratios are in good agreement with the MAX-DOAS values, overall, these findings are much lower than reported by Sinreich et al. (2010), where glyoxal levels were found to reach 140 pptv based on comparable spectroscopic analysis [39]. Satellite measurements made over open tropic waters extrapolated glyoxal concentrations in excess of 200 pptv [34,35], which is inconsistent with atmospheric models that predict trace levels in these isolated regions [9,13,40]. The literature indicates that models underestimate glyoxal production, particularly in urban and remote regions. This underestimation is likely due to the incomplete representation of volatile organic compound (VOC) oxidation pathways in current models, including those from biogenic and anthropogenic sources. Additionally, some studies suggest that secondary organic aerosol (SOA) formation mechanisms, heterogeneous reactions, and glyoxal’s rapid atmospheric removal processes may not be fully captured. Other overlooked aspects include the complexity of multi-phase chemistry and uncertainties in emission inventories, which also contribute to discrepancies between modeled and observed glyoxal levels. In addition, the determination of glyoxal using Fast LED-CE-DOAS, MAX-DOAS, and long-path DOAS faces limitations due to variability in atmospheric conditions, sensitivity, and spatial resolution. Fast LED-CE-DOAS offers high temporal resolution but can suffer from short optical paths, limiting detection sensitivity in low-glyoxal environments. MAX-DOAS and long-path DOAS provide better spatial coverage but are more susceptible to interference from aerosols and other trace gases, which can complicate the accurate retrieval of glyoxal concentrations across varying atmospheric conditions.

In remote ocean measurements, Sinreich et al. (2010) ruled out transport of glyoxal or its precursors from anthropogenic and terrestrial sources due to the long travel time (>1 week) that the air mass required to reach their sampling location 3000 km off the coast [39]. Globally, glyoxal has an atmospheric lifetime of 2–3 h [9,40] based on known sinks due to photolysis, reaction with hydroxyl radicals, and uptake into aerosols [23]. Similarly, isoprene has an estimated lifetime of 1–4 h over the ocean [41,42,43,44]. Taking into account the aforementioned range of glyoxal levels, an average of 50 pptv isoprene in the atmosphere based on a handful of measurements made over ocean waters [45], and an estimation that nearly half of all glyoxal yields from the oxidation of isoprene [9], it has been suggested in numerous manuscripts that there exists a missing source of glyoxal, likely of marine origin, that is at least an order of magnitude greater that what is currently known [9,33,38,39,40].

In recent reports, Zhou et al. (2014) demonstrated that glyoxal can be formed as a secondary or tertiary lipid peroxidation product of polyunsaturated fatty acids (PUFA) [46]. Ozone was made to pass over a monolayer of linoleic acid (C18:2, ∆ 9,12) on artificial seawater or sea surface microlayer (SSML) water collected from the Pacific ocean, which was placed in a flow tube, and its products monitored on-line by proton transfer reaction mass spectrometry. The authors reported a yield of less than 1%, which they attributed to dissolution of glyoxal in the condensed phase due to its highly soluble nature (Henry’s law constant, H_eff_ = 4.19 × 10^5^ M/atm) [47]. Additionally, malondialdehyde, an intermediate to ozone-produced glyoxal, has not been detected in the atmosphere. Based on these findings, the authors suggest that it is unlikely that this chemistry could promote a flux of glyoxal that is able to explain observed atmospheric concentrations.

In this work, however, we used mass spectrometric analyses of nascent sea spray aerosols (SSA) and the SSML of a naturally occurring algal bloom isolated within an ocean-atmosphere laboratory system to show for the first time that appreciable levels of glyoxal can be produced. We provide evidence that, as the algae die during the bloom, their phospholipids are enriched in the SSML, especially within the foam, and undergo autoxidation thereby generating glyoxal as a degradation product. We postulate that the enrichment of organic matter in the SSML creates a hydrophobic environment that affects the solubility of glyoxal such that bubble bursting action from wave breaking facilitates its transport across the air–water interface. Based on these findings, we propose that enrichment occurring during the death phase of an algal bloom and subsequent degradation could potentially serve as an important and currently missing source of glyoxal in the atmosphere.

## 2. Experimental Design

### 2.1. Investigation into Marine Aerosol Chemistry and Transfer Science (IMPACTS)

A natural phytoplankton bloom was initiated on 3 July 2014, in a highly collaborative, month long wave channel experiment, Investigation into Marine Aerosol Chemistry and Transfer Science (IMPACTS). Approximately 13,000 L of seawater was transported from the pumping station approximately 5 m below the sea surface at the Scripps Institution of Oceanography (SIO) pier in La Jolla, CA, USA (32°51′56.8″ N: 117°15′38.48″ W), filtered with a 50 μm mesh to remove debris and zooplankton, and added to a 33 m × 0.5 m channel. Nutrients (Proline A and B, Aquatic Eco-Systems, Apopka, FL, USA) in an F/2 concentration and solutions of sodium metasilicate were added to the channel simultaneously with the seawater to ensure a well-mixed environment. A total of 9 μM sodium phosphate was also added on 25 July 2014. The channel was continuously illuminated in order to promote eukaryotic growth. Chlorophyll *a* measurements recorded by the Southern California Coastal Ocean Observing System’s (SCCOOS, http://www.sccoos.org/) (accessed on 30 June 2014) automated shore station located at the SIO pier confirms that the observed blooms within the wave channel (Figure S1a) were comparable in magnitude to those of the natural environment (Figure S1b). The open wave channel was retrofitted with an air filtration system to create a low background environment (Figure S2). The system can provide particle-free air to the headspace of the channel at an airflow velocity of 12.2 cm·s^−1^. In addition to removing the particulates, the air was scrubbed free of volatile organic compounds (VOCs), nitrogen, and sulfur oxides by passing the air through a four-stage filtration system (Hydrosil International). The filter system consisted of a prefilter, a 4-mm coal-based filter, a pellet-activated charcoal filter, and a potassium permanganate (KMnO_4_) filter (see [48] Supplemental Information). Nascent SSA were generated through the action of breaking waves [48], which, for the purpose of this manuscript, were collected daily for two 2-h periods (4 h total) at ~1 L/min into an impinger containing ~10 mL of 60 mg/mL *o*-phenylenediamine (*o*-PDA, Sigma P23938, St Louis, MI, USA) in 60% tetrahydrofuran (THF)/H_2_O (Figure S2).

**Figure 1:**
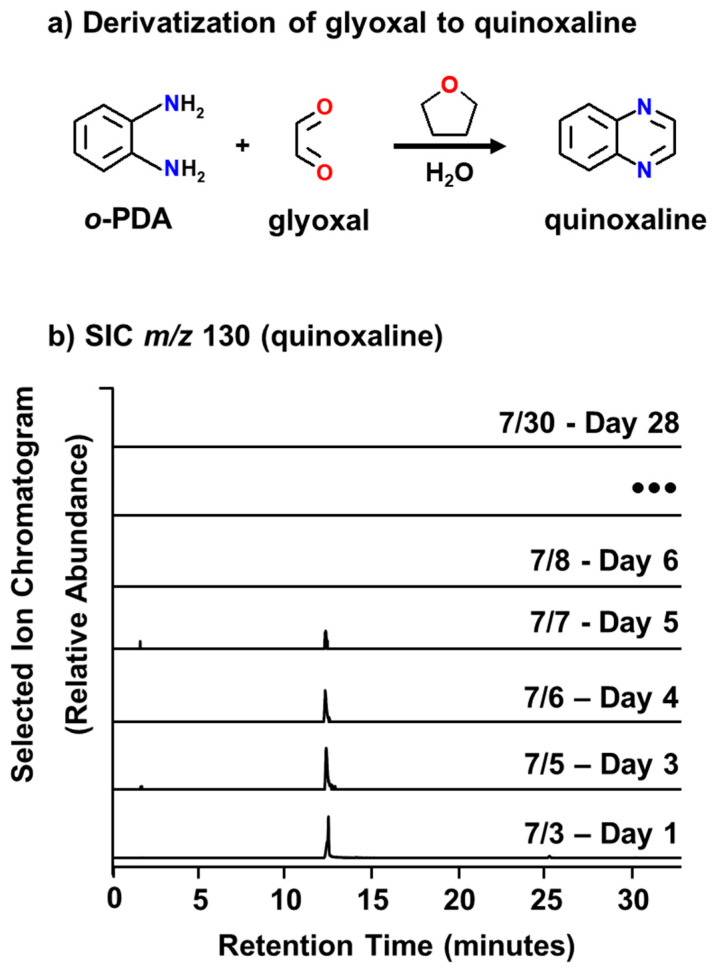
Derivatization of glyoxal to quinoxaline (**a**) for the purpose of GC analysis over the course of IMPACTS (**b**), which was verified by characterization of the retention time and MS spectrum of a commercially purchased standard (Figure S3).

Derivatization of glyoxal to quinoxaline at room temperature (Figure 1a) stabilizes the molecule for analysis, which is an established technique for glyoxal as well as other dicarbonyls [49,50,51]. The mass collected in the impinger from the SSA was concentrated using manual solid phase extraction (Supelco Superclean ENVI-18 SPE, Sigma Aldrich 57064, St Louis, MI, USA) in triplicate, washed with THF/H_2_O to remove unreacted derivatizing agent (because *o*-PDA and quinoxaline co-elute), and then eluted with several column volumes of acetonitrile (ACN). The ACN was removed under vacuum and the samples reconstituted in 500 μL of ACN in preparation for gas chromatography mass spectrometry (GC/MS) analysis. Controls samples within the wave flume at the start of IMPACTS were not possible, given that the timing of the water collection that was pumped into the wave channel corresponded with a stage of the pre-existing natural phytoplankton bloom that was later understood and postulated to be the initiator of algae-derived glyoxal (see Section 3 Results and Discussion). As a substitute, a daily *o*-PDA-doped solid phase microextraction (SPME) fiber was exposed to the room where the sample preparation took place and was transported with the analysis fibers as a control. Additionally, a suite of SSA, SSML, and bulk measurements were made daily by collaborators to monitor the physical, chemical, and biological changes that occurred throughout the course of the channel experiment.

### 2.2. Marine Aerosol Reference Tank (MART)—Mesocosm Scale

For MART details, please see Stokes, M.D. et al. [52]. Ocean surface water was collected by bucket off of the end of the SIO pier, mesh filtered, added to a MART, and supplemented with F/2 media. Upon algal death, foam was collected into a glass jar by pipette from atop the SSML. Once settled, the now liquid mixture was concentrated using SPE (Supelco Superclean ENVI-18 SPE, Sigma Aldrich 57064, St Louis, MI, USA) in triplicate, washed with several column volumes of water to desalt the sample, and then eluted with methanol until all visible pigmentation was removed from the SPE resin bed. The methanol was removed under vacuum and the sample reconstituted in 100 μL of a 5% ACN/H_2_O, vortexed to ensure homogeneous mixing, and then spun down to isolate any insoluble fractions. Molecular characterization of the foam was performed using liquid chromatography high resolution mass spectrometry (LC-HR-MS/MS, Thermo Scientific, Brennen, Germany).

### 2.3. Large Culture Flask with Seawater—Microcosm Scale

Ocean surface water was collected by bucket off the end of the SIO pier and mesh filtered to remove debris and larger microorganisms. A total of 1.5 L of seawater was added to a 2800 mL culture flask and supplemented with inorganic media in excess of F/2 concentration to aid in a rapid bloom cycle. The culture was continuously bubbled with filtered house air and irradiated (254 nm) while gently stirring on an orbital shaker. The SSA and headspace were collected over seven days and analyzed in triplicate with an array of SPME fibers (Supelco PDMS/DVB, 65 μm, Sigma 57326-U; Supelco Carboxen/PDMS-DVB, 85 μm; Supelco PDMS, 100 μm, Sigma 57300-U; and *o*-PDA loaded PDMS/DVB; Sy Louis, MI, USA) as well as bubbled into an impinger of *o*-PDA dissolved in 60% THF/H_2_O. The impinger samples were processed using SPE as previously described herein. All samples were analyzed by GC/MS (Newport, DE, USA).

### 2.4. Flask Experiment with Proxy Molecules—Proof of Concept

Linoleic (C18:2) fatty acid methyl ester (FAMEs) was made from the transesterification of cooking oil (triglycerides) [53]. The FAMEs were purified on a Shimadzu CBM 20A preparatory scale HPLC (10% methanol/ACN, isocratic elution, flow rate = 7.0 mL/min) equipped with a SPD-10A UV detector (λ = 205 nm). The C18:2 standard was evaporated onto the inside surface of a round bottom flask and irradiated (254 nm) in a dose-dependent manner for up to 7 h while collecting the headspace in triplicate on a SPME fiber (Supelco Carboxen/PDMS-DVB, 85 μm, St Louis, MI, USA). The SPME samples were analyzed by GC/MS.

### 2.5. Gas Chromatography Mass Spectrometry (GC/MS)

An Agilent 7820A GC was used for all gas analyses. Impinger samples were introduced to the GC via an autosampler (1 μL) and SPME fibers were manually injected. The samples were desorbed onto a Restek Corporation RTX-5 column (30 m × 250 μm × 0.25 μm; Cat# 10223, Centre County, PA, USA) for separation before detection by the 5975 MSD system. GC/MS parameters for the wave flume impinger samples and the SPME fibers used during the culture flask experiment are as follows: inlet = 175 °C; gradient = 90 °C (3 min), ramped to 100 °C at 0.75 °C/min (hold 2 min), and further ramped to 220 °C at 3 °C/min (hold 7 min); split = 25:1; range = *m*/*z* 45–550; Electrom multiplier Voltage, EMV = 3000; MS source = 230 °C; and MS quadrupole = 150 °C. Headspace collected via impinger and SPME for the proof-of-concept experiments were analyzed as follows: inlet = 175 °C; gradient = 60 °C (1 min) and ramped to 225 °C at 10 °C/min (hold 5 min); split = 100:1; range = 40–400; EMV = 3000; MS source = 230 °C; and MS quadrupole = 150 °C.

### 2.6. Liquid Chromatography High Resolution Tandem Mass Spectrometry (LC-HR-MS/MS)

A 20 μL aliquot of the foam sample was separated on an analytical column [Vydac C4, 1 mm × 150 mm, 5 μm, S/N 041602 p; 80 μL/min; 30 °C; 20% B (1 min) ramped to 100% B (38 min) and hold for 5 min; A = 2% ACN, 0.05% formic acid in water, B = 100% ACN] and the eluent detected by high resolution tandem mass spectrometry in positive-ion data dependent modes on a Thermo LTQ-Orbitrap XL [FTMS scan = 1; *m*/*z* 300–1800; resolution = 15,000; ion trap CID scans = 6; source temperature = 275 °C; ESI voltage = 3.8 kV; capillary voltage = 39 V; tube lens = 135 V; nebulizer gas N_2_ = 38; CID collision energy = 30].

### 2.7. Chlorophyll a Measurements

Bulk chlorophyll *a* concentrations were measured fluorometrically using a Wetlabs ECO BBFL2 sensor (470/695 nm EX/EM, Philomath, OR, USA) and Turner AquaFluor handheld unit (395,130/≥600 nm EX/EM, San Jose, CA, USA). The Wetlabs sensor was integrated into a flow-through system where the water was pumped into an inline fluorimeter and then returned to the enclosed channel system, never being exposed to the atmosphere outside of the wave flume to allow for continuous measurements. AquaFluor was used with discrete samples. The Wetlabs sensor was calibrated at the factory against *T. weissflogii* cultures and used as received. Measurements made with the Turner AquaFluor were calibrated against simultaneous values recorded by the Wetlabs sensor, and the data from both instruments were combined into a single continuous time series.

### 2.8. Lipase (Stearase) Activity Measurements

Lipase activity was measured using the 4-Methyl-umbelliferone (MUF) fluorogenic substrate method [54]. The MUF stearate was added to a final concentration of 20 µM [55]. The samples were incubated at the in situ temperature in the dark for one hour. Assays were performed in microtiter plates (SpectraMax M3, Molecular Device, San Jose, CA, USA) in triplicates. Fluorescence was measured immediately after adding substrates and again at the end of the incubation [56], at 355/460 nm (excitation/emission). The fluorescence signal after blank correction was calibrated against the 4-MUF standards. Stearase activities are reported as nmol substrate hydrolyzed per liter hour (nM/h). The SSML sample was diluted 10 times with a batch of 0.2 µm filtered autoclaved seawater prior analysis.

## 3. Results and Discussion

### 3.1. The IMPACTS Experiment—Investigation into Marine Aerosol Chemistry and Transfer Science (IMPACTS)

To determine whether glyoxal could be generated and transferred to the atmosphere as a result of marine microbiological activity, a mesocom experiment, Investigation into Marine Aerosol Particle Chemistry and Transfer Science (IMPACTS), was performed in a wave channel filled with 13,000 L of seawater and added nutrients in order to promote eukaryotic growth for the purpose of simulating microbiological-driven chemical processes native to ocean waters. As shown in Figure 1b, glyoxal, in the form of quinoxaline, was detected during the first five days of the mesocom experiment. Based on SCCOOS chlorophyll *a* measurements (Figure S1b) this time frame corresponded to the prolonged death phase (1–5 July 2014) of a naturally aged algal bloom that was present along the La Jolla coast at the time of seawater collection (2 July 2014) and the start of data acquisition (3 July 2014).

### 3.2. Verification of IMPACTS at a Microcosm Scale

**Figure 2:**
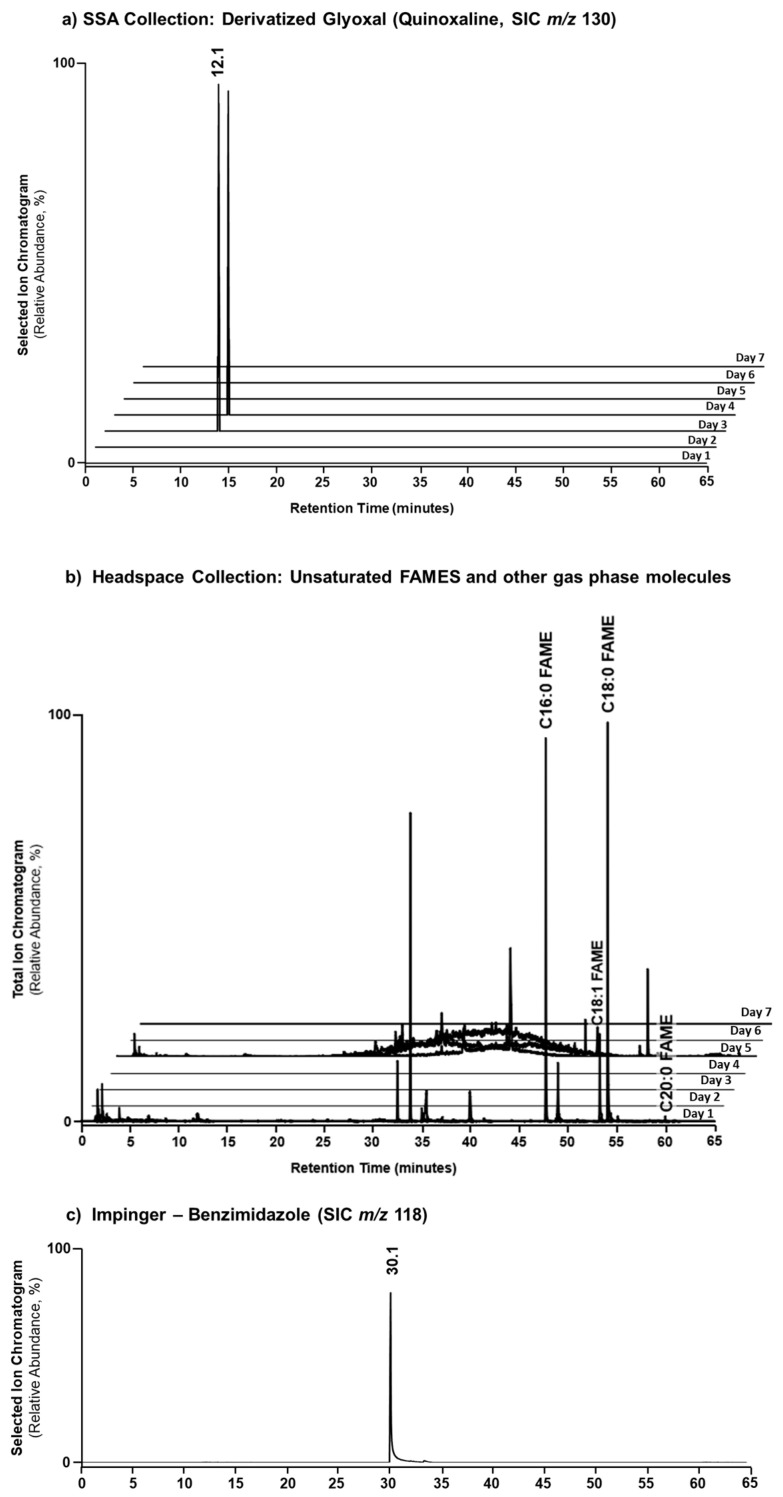
GC/MS temporal chromatograms of glyoxal transported by SSA and collected within an impinger of *o*-PDA where it was derivatized to quinoxaline (RT 12.1; SIC *m*/*z* 130) (**a**); and gas-phase molecules captured by an array of SPME fibers (traces are superimposed) highlighting the primary presence of unsaturated FAMEs (**b**) during the microcosm culture flask experiment. Formaldehyde was also characterized in the impinger of *o*-PDA where it was derivatized to benzimidazole (RT 30.1 min, SIC *m*/*z* 118) during the microcosm experiment. Labeled peaks were verified by retention time and MS spectrum using standards (Figures S3–S5). (**c**) the reaction product of *o*-PDA and formaldehyde was detected, which implies methanol may have undergone oxidation.

To validate that the presence of marine glyoxal is indeed linked to the microbiological activity of the seawater and was not an artifact of the wave channel, a subsequent culture flask experiment was conducted in a fume hood using 1.5 L of SIO pier seawater (collected 8 August 2014) spiked with excess nutrients to aid in a quick bloom cycle. Again, utilizing *o*-PDA derivatization within an impinger and GC/MS analysis, glyoxal was detected on Days 3–4 of the collection period (Figure 2a). Together, these findings definitively demonstrate that the ocean can contribute to the levels of glyoxal found in the atmosphere. Moreover, these data suggest that glyoxal formation is directly linked to the life-cycle of the phytoplankton.

In addition to the SSA collection via impinger, the SSA and headspace of the culture flask experiment were also sampled using a variety of SPME fibers (Figure 2b). The chromatograms for each fiber type used per day were overlayed to provide an aggregated view of the molecules captured. Primarily fatty acid methyl esters (FAMEs) [palmitic (C16:0, RT 47.6 min), stearic (C18:0, RT 53.9 min), oleic (C18:1, RT 53.2 min), and arachidic (C20:0, RT 59.9 min)] were detected during Day 1 and Day 5. Due to the toxic nature of free fatty acids to both eukaryotic and prokaryotic cells [57], fatty acids chains are predominately found in the form of ester- and ether-linkages in cell membrane components such as glycolipids, triacylglycerides, and phospholipids. In the same vein, FAMEs are not typically occurring cellular molecules, although 3-hydroxy C16:0 FAME has been identified as an endogenous volatile extracellular factor in cell density-associated regulation of virulence genes in the plant pathogenic bacteria *Ralstonia solanacearum* [58]. As such, the presence of FAMEs likely indicates the death and cellular decomposition of the phytoplankton, followed by the transesterification of fatty acid containing molecules to yield FAMEs. The presence of FAME implies that methanol is present. The oxidation of methanol produces formaldehyde, although it is possible that formaldehyde is produced in another way. Although it was not directly observed, benzimidazole, the reaction product of *o*-PDA and formaldehyde was detected (Figure 2c), which implies methanol may have undergone oxidation.

### 3.3. SSML Analysis with Marine Aerosol Reference Tank (MART)

**Figure 3:**
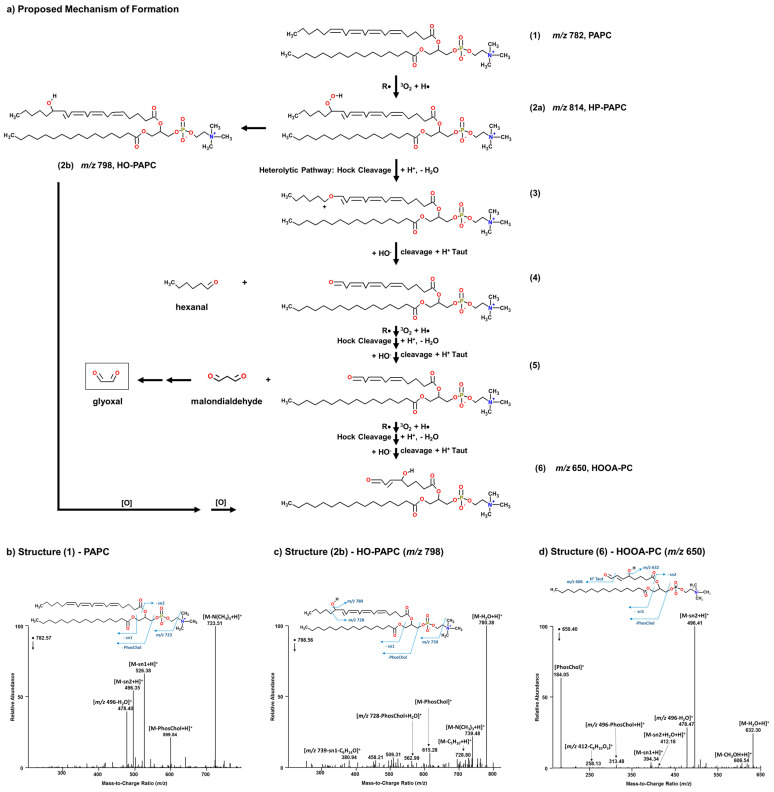
Proposed mechanism for the formation of marine glyoxal from the peroxidation of PUFA-containing phospholipids (**a**), which was validated by the ESI-MS/MS positive-ion mode characterization of PAPC (**b**), its oxidation product HO-PAPC (**c**), and the corresponding short-chain degradation product HOOA-PC (**d**) that were found in the foam of the SSML.

**Figure 4:**
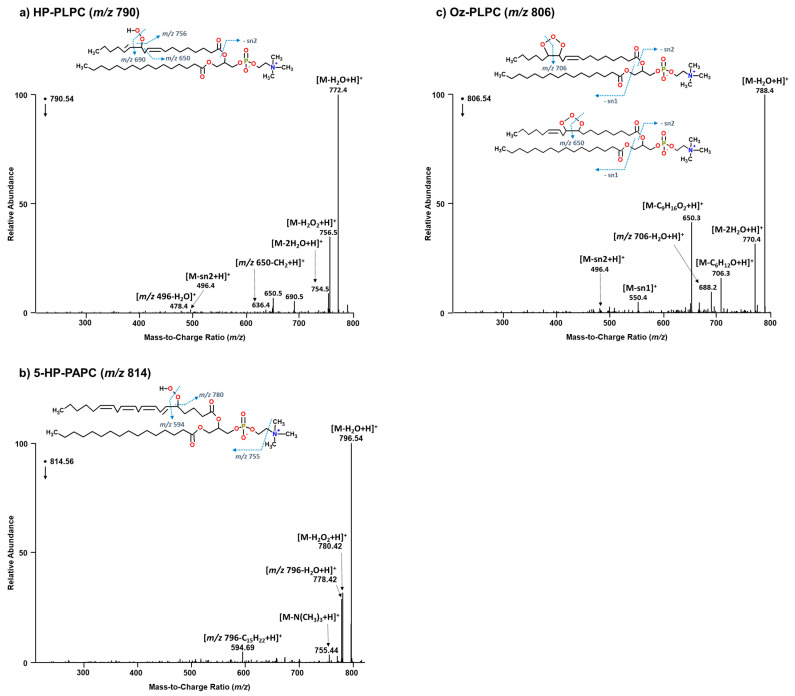
ESI-HR-MS/MS positive-ion spectra of peroxidized PLPC (**a**) and PAPC (**b**); and ozonides of PLPC (**c**).

During a smaller mesocom experiment conducted in a marine aerosol reference tank (MART) [52] prior to the start of IMPACTS, foam was collected from atop the SSML at the end of a phytoplankton bloom and analyzed by LC-HR-MS/MS. Peroxidized phospholipids and their corresponding short-chain oxidation products were identified. Specifically, a series of diacyl phosphatidylcholines were characterized, which are uniquely abundant in eukaryotes, thus allowing for distinction between algal- and bacterial-origins. The presence of these phospholipids suggests a potential mechanism by which marine glyoxal is formed from the oxidation and cleavage of the PUFA. For example, Figure 3a outlines a potential mechanism by which glyoxal can be produced from the oxidation and cleavage of the PUFA chain of 1-palmitoyl-2-arachidonoyl-3-glycerophosphocholine (PAPC). In this mechanism, a radical initiator (R●) is used to represent the activity of a non-ozone oxidizing agent. Upon hydrogen abstraction, triplet oxygen (^3^O_2_) can add to the chain along with a hydrogen atom to form a peroxyl adduct. Heterolytic (Hock) cleavage of the O-O bond occurs resulting in the formation of hexanal. Glyoxal can then be produced as a secondary oxidation product of malondialdehyde upon repeat of the same chemistry. The reaction terminates when allylic hydrogens are no longer available for abstraction. Figure 2 contains the assigned spectra for PAPC (*m*/*z* 782) (b), its hydroxylated form 1-palmitoyl-2-(ω6-hydroxy-arachidonoyl)-3-glycerophosphocholine (HO-PAPC, *m*/*z* 798) (c), and the short-chain oxidation product 1-palmitoyl-2-(5-hydroxy-8-oxo-6-octenoic acid)-3-glycerophosphocholine (HOOA-PC, *m*/*z* 650) (d), which correspond to structures (1), (2b), and (6), respectively, of the proposed mechanism. Although structure (2a) was not found, the presence of (2b), a 1-palmitoyl-2-(ω7-hydroperoxy-linoleoyl)-3-glycerophosphocholine (HP-PLPC; C16:0/C18:2 ∆ 9,12; *m*/*z* 790, Figure 4a) analogue of PAPC, and a 5-HP-PAPC isomer (Figure 4b) confirms that radical initiated oxidation of phospholipids in the SSML can and does occur. Unlike the wave channel, the air that passed over the water surface in the MART was not scrubbed; thus, ozone (and other oxidants) was also present and reacted as evidenced by the C16:0/C18:2 ∆ 9,12 ozonide-containing phosphotidylcholines (Oz-PLPC, *m*/*z* 806) that were characterized (Figure 4c). In this case, the ozonide products likely contributed to the radical initiated mechanism, and this was in keeping with the finding that showed 85–90% of the ozone adduct decomposes into H_2_O_2_ [59]. The absence of glyoxal during the decline of the chlorophyll *a* peaks during IMPACTS suggests that the oxidants needed to promote this chemistry come primarily in the atmosphere not the SSML. These data demonstrate for the first time that more than one oxidation pathway to the formation of marine glyoxal is possible, and that they can occur not only simultaneously, but synergistically within the same system. It should be noted that the presence of 5-HP-PAPC suggests that some oxidation events may occur endogenously. When intracellular peroxidation of PAPC occurs, it does so almost exclusively on C5 for unclear reasons [60]. Its degradation would yield a long-chain PUF aldehyde that would be highly susceptible to further oxidation and decomposition either in the SSML or within the SSA, given its liberation from the charged phosphocholine headgroup [61]. It should also be noted there is some precedence in mammalian cells for hydratase-facilitated oxidation in vivo of 9Z- and 12Z-unsaturated C16 and C18 fatty acids to yield 10-hydroxy and 10,13-dihydroxy derivatives, respectively. However, the enzyme active site is highly selective for non-ester-bound substrates [62].

**Figure 5:**
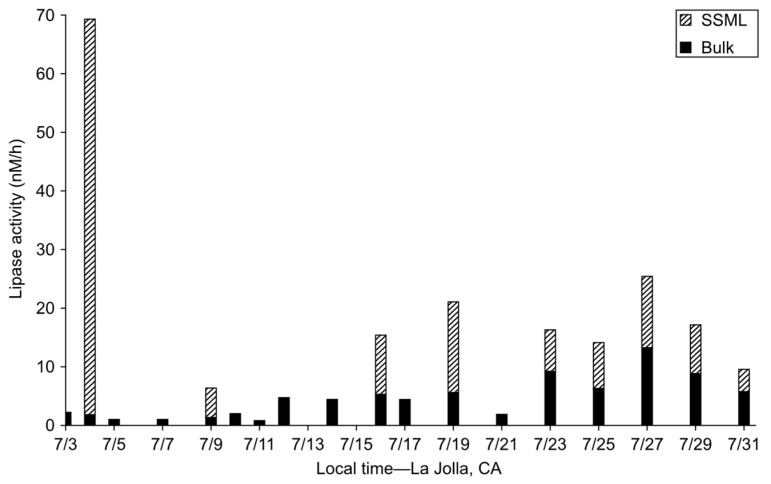
Measured lipase (stearase) activity in the sea surface microlayer (SSML; top of the striped bars) and bulk (top of the solid bars) waters of the wave channel during IMPACTS.

Although the chemistry in Figure 3a is well-supported, the solubility of glyoxal would likely preclude its facile transfer from the aqueous- to the gas-phase as was reported by Zhou et al. (2014) [46]. However, transport across the air–water interface could be facilitated through SSA formation due to the action of bubble bursting from breaking waves. Increased levels of organic carbon (OC) in the SSML are reported to directly correlate with the OC enrichment factor of SSA independent of chlorophyll *a* concentrations [63]. Detection of glyoxal on 3–5 July 2014 may represent that some critical level of OC was achieved that facilitated its enrichment in the SSA, and this happened to correspond with the July 1st chlorophyll *a* peak that likely provided the substrate for glyoxal production. In this vein, a similar trend was observed in the microcosm experiment where there was a burst of OC collected in the headspace on Day 5 (Figure 2b) following glyoxal detection on Days 3–4 (Figure 2a). Further evidence that there were ester-bound lipid substrates present in surface waters at that time comes from the measured enzyme activity within the channel that showed continuous lipase activity in bulk waters with a spike in stearase activity (~70 nM/h), specifically within the SSML on 4 July 2014 (Figure 5). The enrichment of hydrophobic OC (i.e., lipids) and the lipophilic nature of a bacterial cell membrane in the SSML may alter the solubility of molecules such as glyoxal that are formed at the surface in that environment. This may be especially true of the foam, which is highly enriched with organic matter but has a significantly lower water content than the SSML. As a result, glyoxal may be transferred to the atmosphere as OC enriched in the SSA. Despite the uncertainty regarding the exact mode of transport, it is clear that glyoxal may be capable of crossing the air-water interface.

Future work would improve the laboratory studies on the kinetics and pathways of glyoxal uptake and oxidation. The expansion of field campaigns should improve the understanding of glyoxal’s interaction with SSA. The use of multiphase modeling incorporating factors like relative humidity, aerosol composition, and glyoxal’s aqueous-phase reactions will help reduce model uncertainties in predicting its transport and transformation. Lastly, there needs to be a search for a link between glyoxal and secondary organic aerosols.

### 3.4. Gas-Phase Verification of Proposed Mechanism

**Figure 6:**
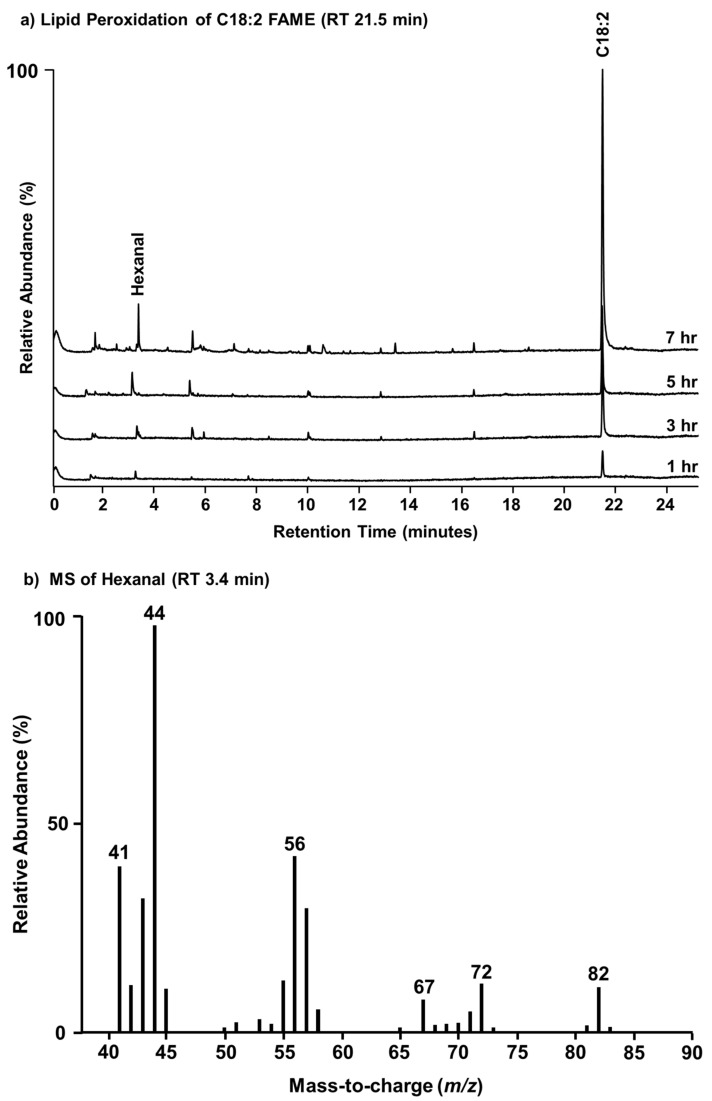
GC/MS headspace analysis of the dose-dependent UV irradiation of C18:2 FAME standard (RT 21.5 min) (**a**) and the MS of hexanal which is an oxidation product of C18:2 (**b**) collected by SPME.

Having observed glyoxal in the form of quinoxaline in SSA during the death phase of a natural algal bloom (IMPACTS mesocosm) as well as in a cultured flask experiment (microcosm), along with characterized oxidized phospholipids and their corresponding short-chain oxidation products in the SSML (MART), it was proposed herein that the formation of marine glyoxal may occur as a degradation product of PUFAs under oxidizing conditions (Figure 3a). To further support this mechanism, a proof-of-concept experiment was carried out to demonstrate that VOCs, such as hexanal, are also formed as proposed and transfer to the atmosphere in the gas phase. To do so, a round bottom flask was coated and dried with an oleic (C18:2) FAME standard, irradiated in a dose-dependent manner with ultraviolet light (254 nm), and the headspace collected by SPME. The GC/MS chromatograms (Figure 6a) and corresponding mass spectrum (Figure 6b) show that hexanal (and heptanal to a slightly lesser extent) is the major degradation product. These findings are consistent with our previously reported observation of hexanal and other VOCs detected in the headspace during the course of a bacteria-promoting seawater culture [59]. Given the use of short-wave UV light in the presence of triplet oxygen (^3^O_2_), ozone was a likely oxidant in this proof-of-concept experiment as previously described [46].

## 4. Conclusions

This work represents the first measurements of glyoxal made on nascent SSA generated during a naturally occurring phytoplankton bloom within an ocean-atmosphere laboratory system. The data herein unequivocally show that the ocean can serve as a source of glyoxal in the atmosphere due to radical and ozone-initiated oxidation of phospholipids present in the SSML, especially the foam. These findings strongly suggest that glyoxal production is linked to marine microbiological activity based on the following proposed mechanism: Inorganic nutrients drive an algal bloom. When depleted, the phytoplankton die and become organic nutrients for the bacteria, which facilitate the decomposition of the algal cellular structure. As the membrane degrades, its phospholipids are enriched at the air–water interface due to the hydrophobic nature of the fatty acid chains. Given that algal phospholipids are rich with PUFAs, they are highly susceptible to oxidation. Degradation of the enriched oxidized phospholipids yield glyoxal along with other VOCs. Glyoxal transfer from the ocean into the atmosphere is aided by bubble bursting from breaking wave action and likely a critical OC level that changes the solubility conditions at the surface. Thus, the prolonged death phase of an algal bloom serves as an important and currently missing source of marine glyoxal in the atmosphere. This proposed mechanism provides a possible explanation for the large discrepancies in reported glyoxal concentrations measured over open waters.

## Data Availability

The data presented in this study are available on request from the corresponding author. The data are not publicly available due to privacy.

## Supplementary Materials

The following supporting information can be downloaded at: https://www.mdpi.com/article/10.3390/metabo14110639/s1, Figure S1. Phytoplankton bloom conditions during IMPACTS (a), and in the coastal waters off of the SIO pier in La Jolla, CA, Figure S2. Schematic of the air scrubbing system and an impinger for the wave channel during IMPACT, Figure S3. GC/MS chromatogram of a quinoxaline standard, Figure S4. GC/MS chromatogram of FAME standards, Figure S5. GC/MS chromatogram of a benzimidazole standard, Figure S6. Equipments.

## Abbreviations

1-palmitoyl-2-(5-hydroxy-8-oxo-6-octenoic acid)-3-glycerophosphocholine (HOOA-PC); 1-palmitoyl-2-(ω6-hydroxy-arachidonoyl)-3-glycerophosphocholine (HO-PAPC); 1-palmitoyl-2-(ω7-hydroperoxy-linoleoyl)-3-glycerophosphocholine (HP-PLPC); 1-palmitoyl-2-arachidonoyl-3-glycerophosphocholine (PAPC); 4-Methyl-umbelliferone (MUF); acetonitrile (ACN); fast light-emitting diode cavity-enhanced differential optical absorption spectroscopy (Fast LED-CE-DOAS); fatty acid methyl esters (FAME); fatty acids (FA); gas chromatography mass spectrometry (GCMS); hydrogen peroxide (H_2_O_2_); Investigation into Marine Aerosol Chemistry and Transfer Science (IMPACTS); liquid chromatography high resolution mass spectrometry (LC-HR-MS/MS); liquid chromatography tandem mass spectrometry(LC MS/MS); Marine Aerosol Reference Tank (MART); marine boundary layer (MBL); multiaxis (MAX); o-phenylenediamine (o-PDA); organic carbon (OC); ozonide-containing phosphotidylcholines (Oz-PLPC); parts per trillion (pptv; pmol/mol); polyunsaturated fatty acids (PUFA); potassium permanganate (KMnO_4_); radical initiator (R●); Scripps Institution of Oceanography (SIO); sea spray aerosols (SSA); sea surface microlayer (SSML); secondary organic aerosols (SOA); solid phase extraction (SPE); solid phase microextraction (SPME); Southern California Coastal Ocean Observing System’s (SCCOOS); tetrahydrofuran (THF); ultraviolet (UV); volatile organic compounds (VOCs).
