# Supplementary material for: Possible Missing Sources of Atmospheric Glyoxal Part I: Phospholipid Oxidation from Marine Algae
Source: Metabolites. 2024 Nov 19;14(11):639. doi: 10.3390/metabo14110639 (PMC11596397; doi:10.3390/metabo14110639)
Supplement: Supplementary file 1 [file metabolites-14-00639-s001.zip › metabolites-3263005-supplementary.pdf]

metabolites

Supporting Information

# Possible Missing Sources of Atmospheric Glyoxal Part I: Phospholipid Oxidation from Marine Algae

**Renee T. Williams [1], Annika Caspers-Brown [1], Camille M. Sultana [1], Christopher Lee [1], Jessica L. Axson [1,2], Francesca Malfatti [3,4], Yanyan Zhou [4,5], Kathryn A. Moore [1], Natalie Stevens [1], Mitchell V. Santander [1], Farooq Azam [2], Kimberly A. Prather [1,4] and Robert S. Pomeroy [1,*]**

[1] Department of Chemistry and Biochemistry, University of California, La Jolla, San Diego, CA 92093, USA; rwill008@gmail.com (R.T.W.); acaspersbrown@gmail.com (A.C.-B.)
[2] School of Public Health, University of Michigan, Ann Arbor, MI 48109, USA; fazam@ucsd.edu
[3] National Institute of Oceanography and Experimental Geophysics, 34100 Trieste, Italy
[4] Scripps Institution of Oceanography, University of California, La Jolla, San Diego, CA 92093, USA; zhouyyahgy@scsio.ac.cn
[5] State Key Laboratory of Marine Environmental Science, Key Laboratory of the MOE for Coastal and Wetland Ecosystems, School of Life Sciences, Xiamen University, Xiamen 361102, China
* Correspondence: rpomeroy@ucsd.edu; Tel.: +1-858-822-5736

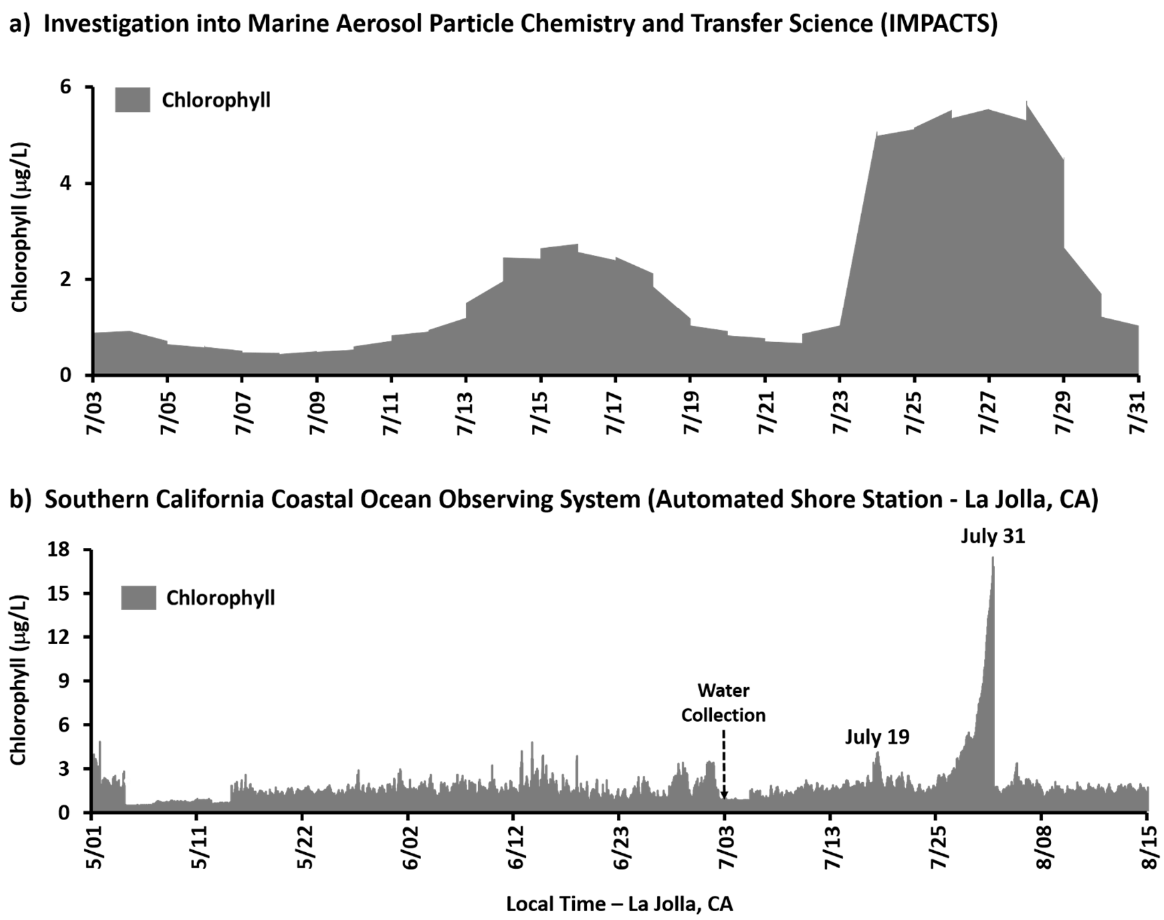


**Figure S1.** Phytoplankton bloom conditions during IMPACTS (a), and in the coastal waters off of the SIO pier in La Jolla, CA (b).

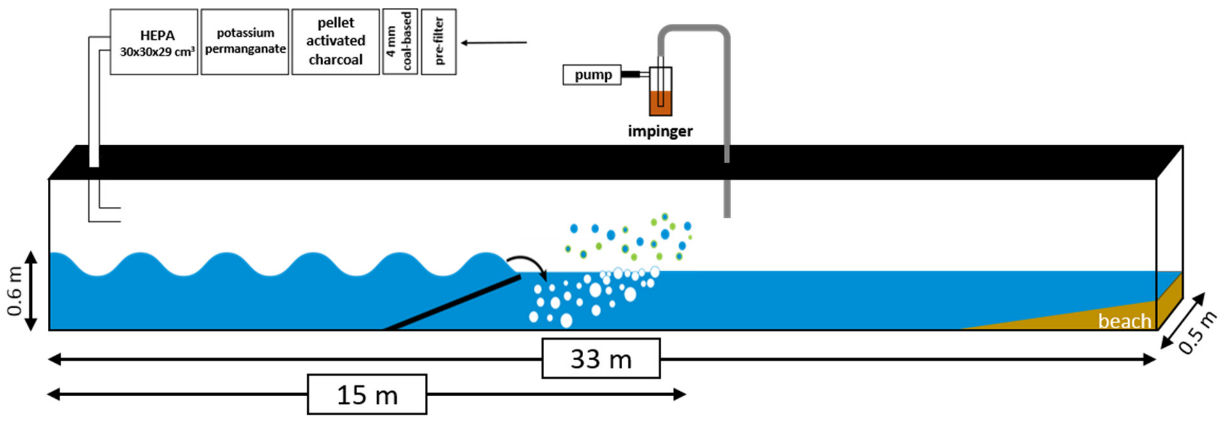


**Figure S2.** Schematic of the air scrubbing system and an impinger of *o*-PDA for collection and derivatization of dicarbonyls as part of the set-up for the wave channel during IMPACT.

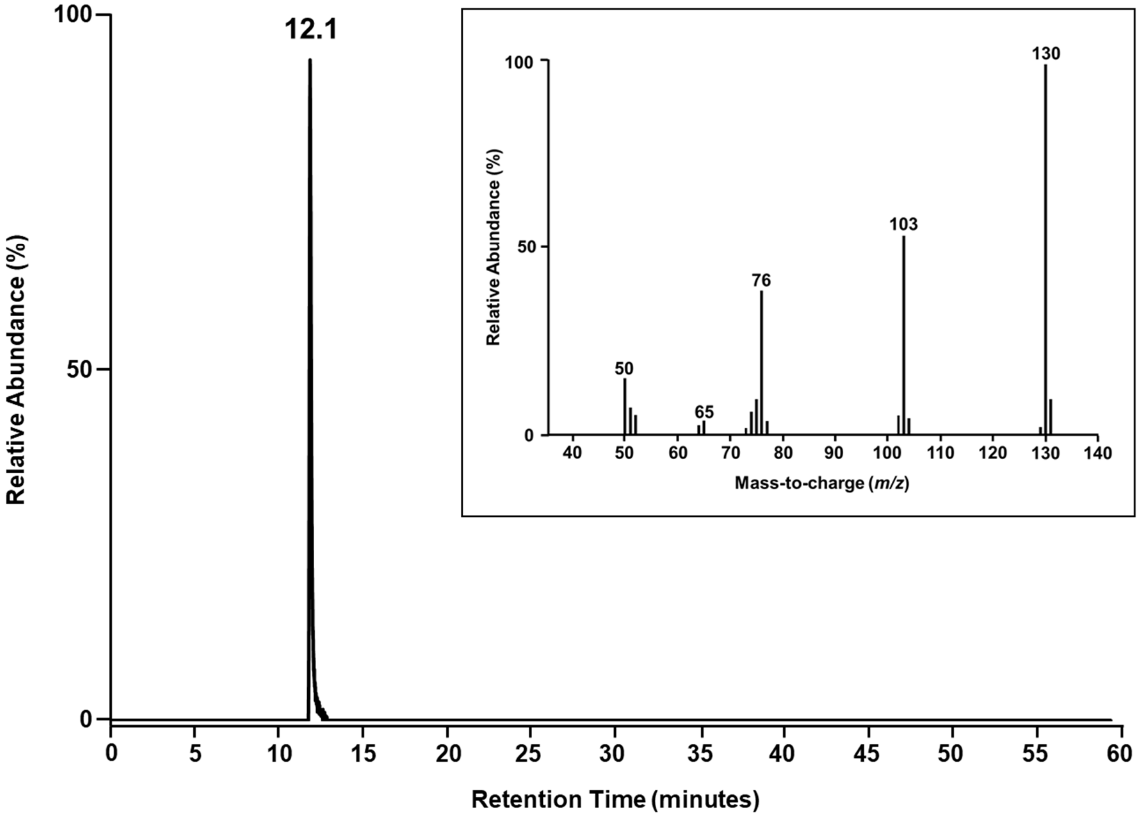


**Figure S3.** GC/MS chromatogram of a quinoxaline standard, which represents the derivatization product of glyoxal and *o*-phenylenediamine (o-PDA) that was used in this manuscript to stabilize glyoxal for offline measurements. Inset is the electron ionization spectrum for quinoxaline (RT 12.1).

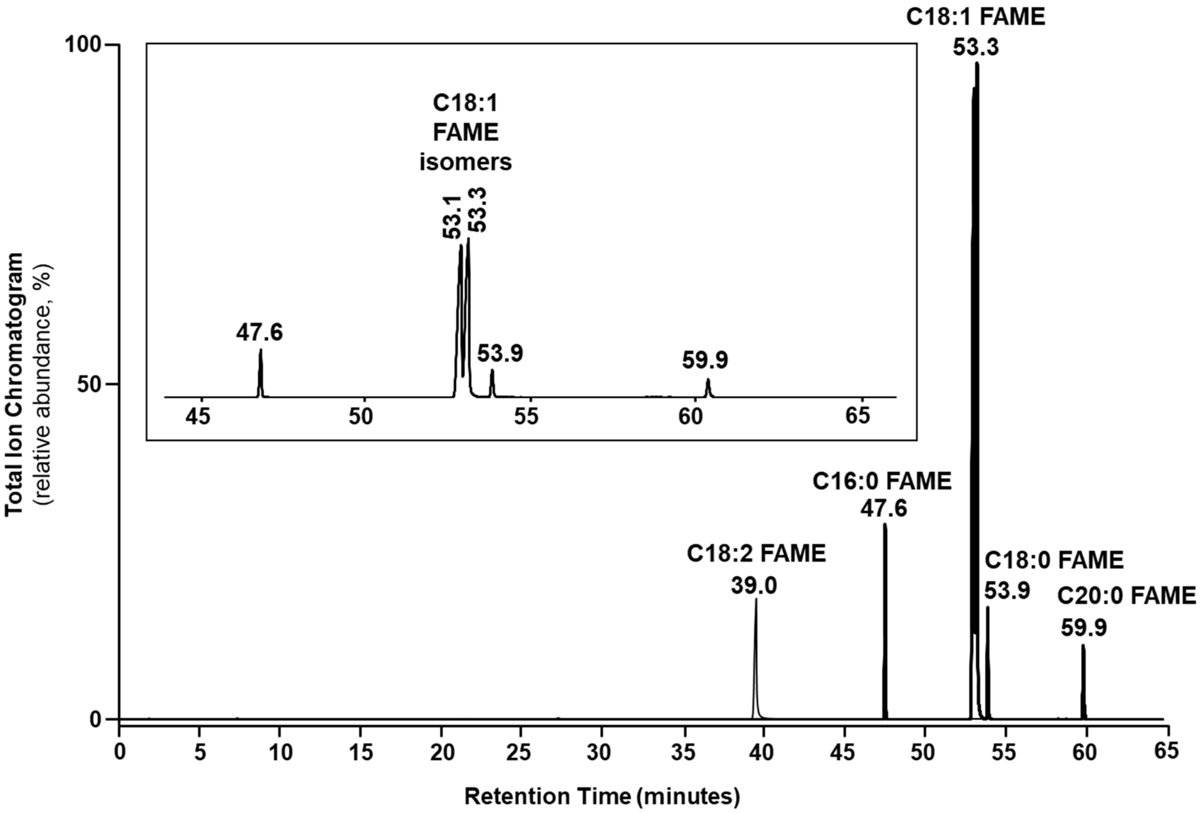


**Figure S4.** GC/MS chromatogram of FAME standards on a non-polar stationary column (Restek Corporation RTX-5): linoleic (C18:2, RT 39.0 min), palmitic (C16:0, RT 47.6 min); stearic (C18:0, RT 53.9 min), oleic (C18:1, RT 53.2 min), and arachidic (C20:0, RT 59.9 min)]. Inset highlights the isomer peaks of oleic FAME.

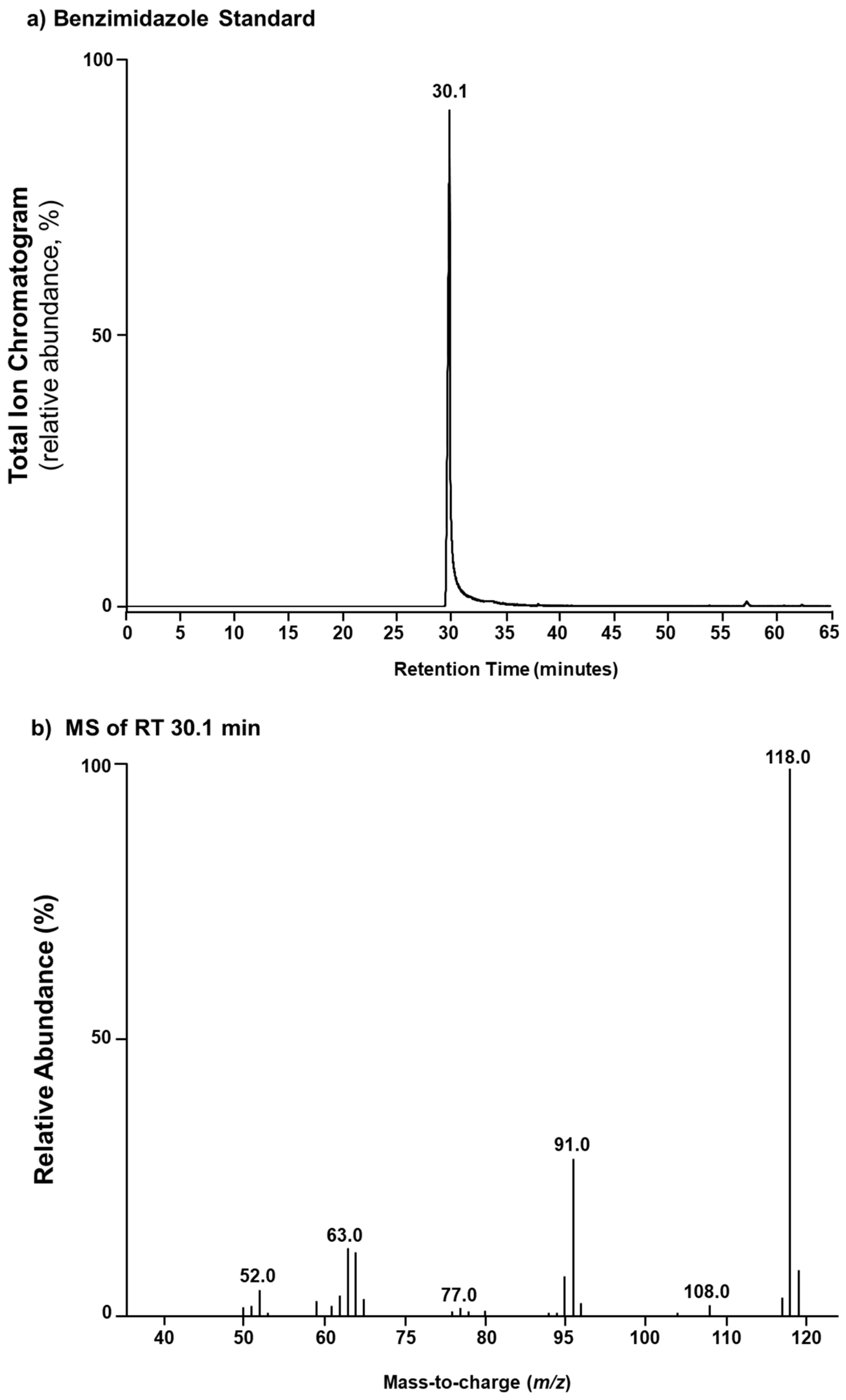


**Figure S5.** GC/MS chromatogram of a benzimidazole standard (RT 30.1 min) (a), and the corresponding electron ionization spectrum (b).

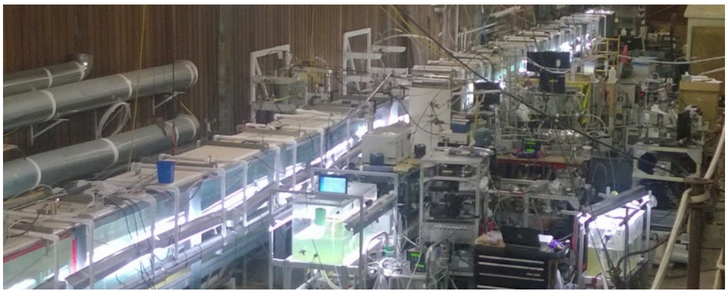

(a) IO Ocean-Atmosphere Facility Hydraulics Laboratory. Wave channel during IMPACTS.

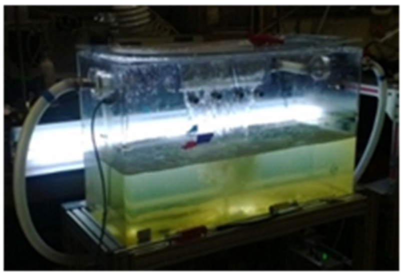

(b) Marine Aerosol Reference Tank (MART).

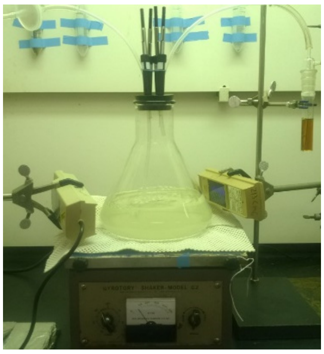

(c) Large Culture Flask Set-Up.

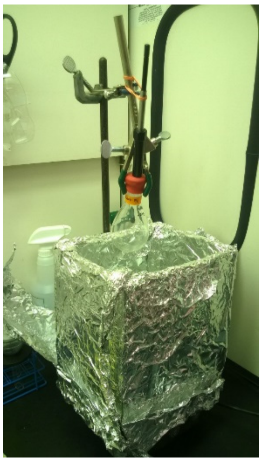

(d) Small Flask Irradiation Set-Up.

**Figure S6.** Equipments (a) IO Ocean-Atmosphere Facility Hydraulics Laboratory. Wave channel during IMPACTS. (b) Marine Aerosol Reference Tank (MART). (c) Large Culture Flask Set-Up. (d) Small Flask Irradiation Set-Up.
